# Reply: Capecitabine and bevacizumab as first-line treatment in elderly patients with metastatic colorectal cancer

**DOI:** 10.1038/sj.bjc.6606043

**Published:** 2010-12-14

**Authors:** J Feliu, J Barriuso

**Affiliations:** 1 Medical Oncology Service, Hospital La Paz/Autónoma University School of Medicine, IdiPAZ, RETIC, P° de la Castellana, 261- 28046 Madrid, Spain

**Sir,**

I thank Drs Molina-Garrido and Guillén-Ponce (Molina-Garrido and Guillén-Ponce, 2010) for their kind commentaries that we share almost completely.

It is obvious that, as oncologists, we need a tool that allows us to identify those elderly patients who are more sensitive to chemotherapy and are therefore more prone to developing complications resulting from cancer treatments. Nowadays, it is considered that comprehensive geriatric assessment (CGA) is more effective than the standard medical evaluation in the elderly (Gosney, 2005). This tool was designed to evaluate the following aspects: functional status, comorbidity, mental status, emotional conditions, polypharmacy and geriatric syndromes (Balducci and Extreman, 2000; Rao *et al*, 2004). The use of this scale allows us to divide patients into three different groups from a therapeutic point of view: fit (same treatment strategies as those used in younger patients), frail (reduced dose or no chemotherapy) and intermediate. Patients of the first two groups are easily classified. However, the intermediate group is more difficult to identify (Köhne *et al*, 2008). Notwithstanding the multidimensional character of the CGA scale, only two parameters’ comorbidity and performance status (measured using the Lawton–Brody scale and the Barthel scale) are used to distinguish between the fit and the intermediate groups (Balducci and Extreman, 2000; Köhne *et al*, 2008). We used these two parameters alone because of this later fact as inclusion. Nevertheless, each investigator could decide to use the CGA scale following its own criteria or depending on patient characteristics.

It must be said that even if the CGA scale is a useful tool, several authors have described some limitations (Carreca *et al*, 2005): it is not standardised, it is time consuming and it is fragmentary, thereby impeding a correct global assessment of the patient. These limitations have prevented its general use. Therefore, during the last few years, several authors have made an effort to simplify the CGA scale to transform it into a more practical tool. However, most of these proposals were developed after this study was designed. These studies have shown similar outcomes between the CGA scale and the new tools. Nevertheless, it is essential to study the capacity of these new tools to identify vulnerable patients with trials specifically designed for this purpose.

There is a long road to walk in the field of elderly cancer patients. Unfortunately, as far as medical oncologists are bad geriatricians and as geriatricians are not good oncologists, a multidisciplinary collaboration is necessary to improve the treatment of elderly patients with cancer.
